# Robot-Assisted Thoracolumbar Fixation After Acute Spinal Trauma: A Case Series

**DOI:** 10.7759/cureus.31832

**Published:** 2022-11-23

**Authors:** Lance M Villeneuve, Benjamin Lee, Benjamin Cornwell, Murali Nagarajan, Zachary A Smith

**Affiliations:** 1 Department of Neurosurgery, University of Oklahoma Health Sciences Center, Oklahoma City, USA; 2 Department of Radiological Sciences, University of Oklahoma Health Sciences Center, Oklahoma City, USA

**Keywords:** robot-assisted surgery, thoracic spine fracture, thoracolumbar spine, spine trauma and disease, robotic spine surgery

## Abstract

Background: Pedicle screw fixation has become the workhorse for the stabilization of the thoracolumbar spine. Since accurate pedicle screw placement is necessary for a successful surgery, three-dimensional navigation has become a mainstay for placing pedicle screws. However, the published studies have an overrepresentation of lumbar screws despite the prevalence of thoracic fractures. Furthermore, no robotic-assisted pedicle screw study has focused solely on traumatic fractures. The goal of this study was to address whether (1) robot-assisted pedicle screw placement had comparable accuracy in the thoracic and thoracolumbar region and (2) robot-assisted spine surgery was feasible in an acute, traumatic setting.

Methods: We performed 14 consecutive, thoracolumbar spinal stabilization procedures in which 126 pedicle screws were placed using the Globus ExcelsiusGPS® spine robot in an acute, traumatic setting. Operative times were measured, and the accuracy of pedicle screws was assessed with the Gertzbein and Robbins classification system by two board-certified neuroradiologists.

Results: A total of 60-thoracic (T3-T11), the 24-thoracolumbar junction (T12-L1), 40-lumbar (L2-L5), and two-sacral pedicle screws were placed. Pedicle screw placement was accurate with a < 1% (1/126) pedicle breach rate. Thoracolumbar robotic spine surgery in an acute, traumatic setting was demonstrated to have a good safety profile with only one minor neurological deficit which was related to positioning. Furthermore, surgical times were inversely related to the case number.

Conclusions: These results together suggest that robot-assisted spine surgery is accurate in the thoracic spine. Furthermore, placement of thoracolumbar screws in an acute trauma is non-inferior to other methods when based on accuracy.

## Introduction

Over 160,000 spine traumas occur yearly in North America with over 90% of those being localized to the thoracolumbar spine [1,2]. Factors increasing the rates of fracture include risky behavior, lower bone density, alterations in vertebral biomechanical range, and increased falls [3-6]. When necessary, operative intervention utilizing pedicle screws has become the treatment of choice for thoracolumbar spine stabilization. During thoracolumbar fusion procedures, minimizing the risk of screw pullout and strength are important modifiable factors. While screw strength is proportional to the inner (minor) screw diameter, the outer (major) diameter is proportional to screw pullout resistance. A common modifiable factor maximizing screw strength and screw pullout resistance is the accurate placement of the thoracic screw in pedicles in addition to the intrinsic properties of the screw [7].

Thoracic spine pedicle fixation can be particularly difficult due to the kyphosis in the thoracic spine producing vertebra-specific optimal trajectories [8]. Robot-assisted pedicle screw fixation seems ideal in the thoracic scenario due to the previously demonstrated superb accuracy [9,10]. However, most published robot-assisted pedicle screw reports are heavily biased toward lumbosacral fusions. In our own data, only 163/402 of the screws placed with robot assistance have been in the thoracic area (unpublished data). Other studies either report an over representation of lumbar pedicle screws [9,10] or have no data on the instrumented level [11]. Such information will lead to an underrepresentation and possibly inaccurate picture of thoracic robot-assisted pedicle screw placement.

Additionally, despite spinal trauma accounting for a significant portion of potential spinal procedures, robot-assisted surgery remains largely untested in acute, spinal trauma. In a sample of studies with a large number of robot-assisted pedicle screws, trauma only represented a small number of the cases representing 0% up to 18% of cases [10,11]. This small representation of trauma cases in large studies dilute the statistics and impede proper conclusion about acute, traumatic cases. Furthermore, the timing for these trauma surgeries is unclear in many studies as timing for trauma surgeries was outside the scope of the papers. Understanding the feasibility of robot-assisted pedicle screw placement in acute trauma is of the utmost importance as recent research has suggested early decompression (<8 hours) and instrumentation after a spinal cord injury is optimal [12,13].

Despite the evidence emphasizing the accuracy of robot-assisted spine surgery, the adoption of these techniques has been slow. Reasons for not using a robot include lack of accessibility to a robot, familiarity with older techniques, and increased setup time. However, these disadvantages need to be balanced against the advantages of robot-assisted spine surgery including dual point registration/verification, a rigid arm to help support, and guide tools, which translate to less deformity on the unstable spine, and pre-operative planning with intraoperative plan modification. These factors have led robotic-assisted spine surgery to be more accurate than other methods outside of acute spine traumas [14] and may lead to increased fusion rates with improved outcomes in the acute, traumatic setting as has been demonstrated outside of acute, traumatic settings [15].

Given the advantages of navigated, robot-assisted surgery, we performed a case series to determine the feasibility of robot utilization for stabilization of acute, thoracolumbar spine injuries. We investigated the accuracy of a robotic system, the amount of blood loss, the length of hospital stays, and the duration of surgery to address the feasibility of robot-assisted spine surgery in a thoracic-biased setting of an acute, traumatic setting. We hypothesized that robot-assisted pedicle screw fixation would be non-inferior to other pedicle screw fixation techniques.

## Materials and methods

This study is a case series of acute, thoracolumbar spinal fracture patients collected from May of 2020 to February of 2022 at a single institution. Each patient was evaluated by a neurosurgeon to determine the individual’s pathology as well as the best operative course. Patients were taken to the operating room within 24 hours of presentation. All surgeries were performed by a single neurosurgeon with resident assistance. All thoracolumbar pedicle screws were planned and placed using a robotic navigational system (ExcelsiusGPS®, Globus, Audubon, PA). Chart reviews were conducted for demographics, operative reports, and perioperative complications. Post-operative CT scans were obtained either intraoperative or post-operatively. The post-pedicle screw placement scans were evaluated in the axial and sagittal cuts for screw breaches in Centricity imaging software by two board-certified neuroradiologists. Any difference of opinion on pedicle screw breach was discussed among the radiologists until a consensus was reached independently of the surgeons. The cut with the largest deviation was measured by the neuroradiologists. The Gertzbein and Robbins system (GRS) was utilized for grading the pedicle screw breaches. A screw was considered accurate if the screw had an acceptable trajectory (in the pedicle or with an in-out-in trajectory) without a neurological deficit. Operative times were defined as when the time of the initial incision to the closure of the skin. Blood loss was estimated for each procedure by the attending neurosurgeon. This study was approved via the University of Oklahoma Health Sciences Center Institutional Review Board (2/2020/IRB #11641).

Workflow

After a thin-cut (0.65-mm) CT scan was obtained, screw trajectories were planned in all three planes and loaded into the ExcelsiusGPS® system. Patients were sedated, intubated by the anesthesiologist, and positioned on an open Jackson table. The patients were prepped and draped. The area of surgery was identified via fluoroscopy. The dissection of the bony elements surrounding the corresponding level of injury was performed. The surveillance frame was clamped to a spinous process. A second surveillance point was placed into the PSIS through a stab incision. Anterior/posterior and lateral images were obtained using a special attachment with a C-arm x-ray machine. Close attention was paid to the stability of the clamp to optimize navigation and the merging of pre-operative and intraoperative images. A breath hold was utilized during imaging based on the utility of the breath hold in unpublished experience. The CT-planned images were merged with the pre-operative radiographs. The ghost construct was verified by the surgeon ensuring the screw trajectories were merged appropriately with the fluoroscopic images using the merge scores. Landmarks were verified before proceeding. The robot was draped in a sterile fashion and brought into the field. The robot arm was aligned with the potential screw hole. The hole was drilled and tapped with concurrent breath holds. The corresponding pedicle screw was placed. Intraoperative neuromonitoring was conducted on all surgeries. After the screws were placed, the corresponding fusion was performed as well as any additional procedures.

## Results

There were 14-acute, spinal column trauma patients who were included in this single-center study for a total of 126 pedicle screws (Table 1).

**Table 1:** Complete list of patients. A complete list of patients and individual patient characteristics. IOI denotes an in-out-in trajectory.

Patient number	Height (m)	Weight (kg)	BMI	Age	Injury	AO Spine Classification	Glascow coma scale	ASIA Scale	Neurological deficit Pre-op	Neurological deficit post-op	Reoperation (Y/N)	No. of Breaches	Location of breach	Gertzbein-Robbins	Highest Dev (mm)	Screw #	Additional procedures	Duration (min)	#levels	Levels fused	Time/level (min)	Blood Loss (mL)	Length of Hospital Stay (days)
1	1.7	79.0	27.34	26	Left L3 facet with superior endplate fracture	B2N0	15	E	None	None	N	0				8	Traumatic CSF leak repair	283	5	L1-L5	56.6	200	7
2	1.9	70.0	20.02	69	L1 Burst	A4N3	15	D	BLE HF 4+/5	Intact	N	0				8	Laminectomy	244	5	T11-L3	48.8	100	5
3	1.6	90.7	35.43	54	L1 Burst	A4N0	15	E	None	None	N	0				8	Laminectomy	268	5	T11-L3	53.6	20	10
4	1.7	85.1	31.26	60	L1 Burst	A3N0	15	E	None	None	N	2	R T12 pedicle breach (5mm/IOI) / L L2 Anterior cortex breach (2 mm)	D, NA	5, 2	8	Laminectomy	340	5	T11-L3	68.0	100	5
5	1.5	77.2	33.41	41	T8 Chance	B1N0	15	E	Hand paresthesia	None	N	1	L T6 pedicle breach (2mm)	B	2	12	None	316	7	T6-L1	45.1	50	9
6	1.7	83.1	27.77	75	T4/5 Chance	B2N0	15	E	None	None	N	0				8	None	307	5	T3-T7	61.4	500	20
7	1.5	54.0	23.37	27	T12 Burst	A3N0	15	E	None	None	N	0				10	Laminectomy, drainage of EDH	217	5	T10-L2	43.4	250	7
8	1.7	104.0	35.15	27	L4 Burst	A3N0	15	E	None	None	N	0				8	Laminectomy	202	5	L2-S1	40.4	300	6
9	1.6	58.9	23.01	62	T12 superior endplate fracture, L1 burst	A4N0	15	E	None	Thigh paresthesia	N	1	L T11 pedicle breach (3mm/IOI)	B	3	10	Laminectomy	261	6	T10-L3	43.5	200	12
10	1.6	55.1	22.35	23	L3 burst	B2N0	15	E	None	None	N	1	L T12 anterior cortex breach (2mm)	NA	2	10	Laminectomy	227	5	T12-L4	45.4	200	8
11	1.6	61.5	23.15	50	T6/7 disc	Not applicable	15	D	BLE paresthesia	BLE paresthesia	N	0				4	Corpectomy	226	3	T6-T8	75.3	150	5
12	1.8	75.0	24.49	65	T12, L1 burst	A4N0	15	E	None	None	N	0				12	Laminectomy	336	7	T9-L3	48.0	300	64
13	1.9	97.8	28.58	63	T8 burst	B1N0	15	E	None	None	N	4	R T6 pedicle breach (4mm/IOI) / R T7 pedicle breach (2mm/IOI) / L T7 pedicle breach (3mm/IOI) / L T10 pedicle breach (2mm/IOI)	C,B,C,B	4,2,3,2	8	Laminectomy	173	5	T6-T10	34.6	100	6
14	1.8	85.7	27.05	88	T9-T10 Hyperextension	B3N3	15	E	RLE 4/5	None	N	0				12	Laminectomy	157	6	T7-12	26.2	200	25

A total of 60-thoracic (T3-T11), 24-thoracolumbar junction (T12-L1), 40-lumbar, and two-sacral pedicle screws were placed. The most common indication for surgery was a burst fracture. The demographics for the patient population are summarized in Table 2 and the characteristics of the procedures are summarized in Table 3. The average blood loss was calculated to be 191 mL with the average hospital stay being 13.5 days.

**Table 2:** Patient demographics.

Parameter	Overall
Number of patients	14
Gender	
Female, n (%)	7 (50%)
Male, n (%)	7 (50%)
Mean age (years)	52.1 ± 19.8
Mean mass (kg)	76.9 ± 15.0
Mean BMI	27.3 ± 4.8

**Table 3:** Procedure characteristics.

Parameter	Overall
Indication for surgery, n (%)	
Burst fracture	9 (64%)
Chance fracture	2 (14%)
Hyperextension	1 (7%)
Facet fracture	1 (7%)
Traumatic disc	1 (7%)
Number of levels treated, n (%)	
3	1 (7%)
4	0 (0%)
5	9 (63%)
6	2 (14%)
7	1 (7%)
8	1 (7%)
Mean number of screws per case, n	9.0 ± 2.1
Mean surgery time (min)	254.1 ± 55.8
Mean blood loss (mL)	190.7 ± 119.1
Mean post-operative stay (days)	13.5 ± 15.1

**Figure 1:**
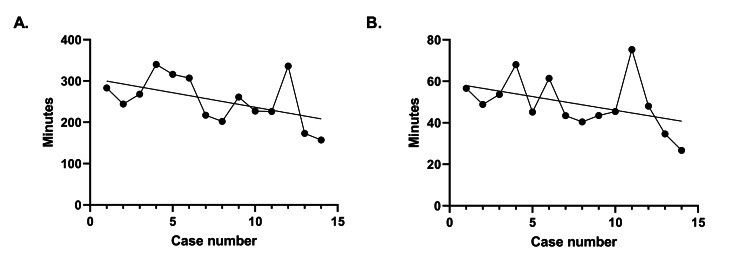
Operative time of surgeries by case number for (A) total operative time and (B) operative time per level. Operative times include any intervening procedures such as laminectomies.

Twenty-six percent (4/15) of patients had pre-operative deficits with three out of four improving after surgery (Table 1). One person had new, post-operative thigh tingling in the lateral femoral cutaneous nerve distribution, (1/14) which was related to positioning and resolved over time. Operative time was graphed against case number which demonstrated the total amount of operative time and the amount of operative time per level that decreased as the surgeon became more experienced (Figure 1).

**Figure 2:**
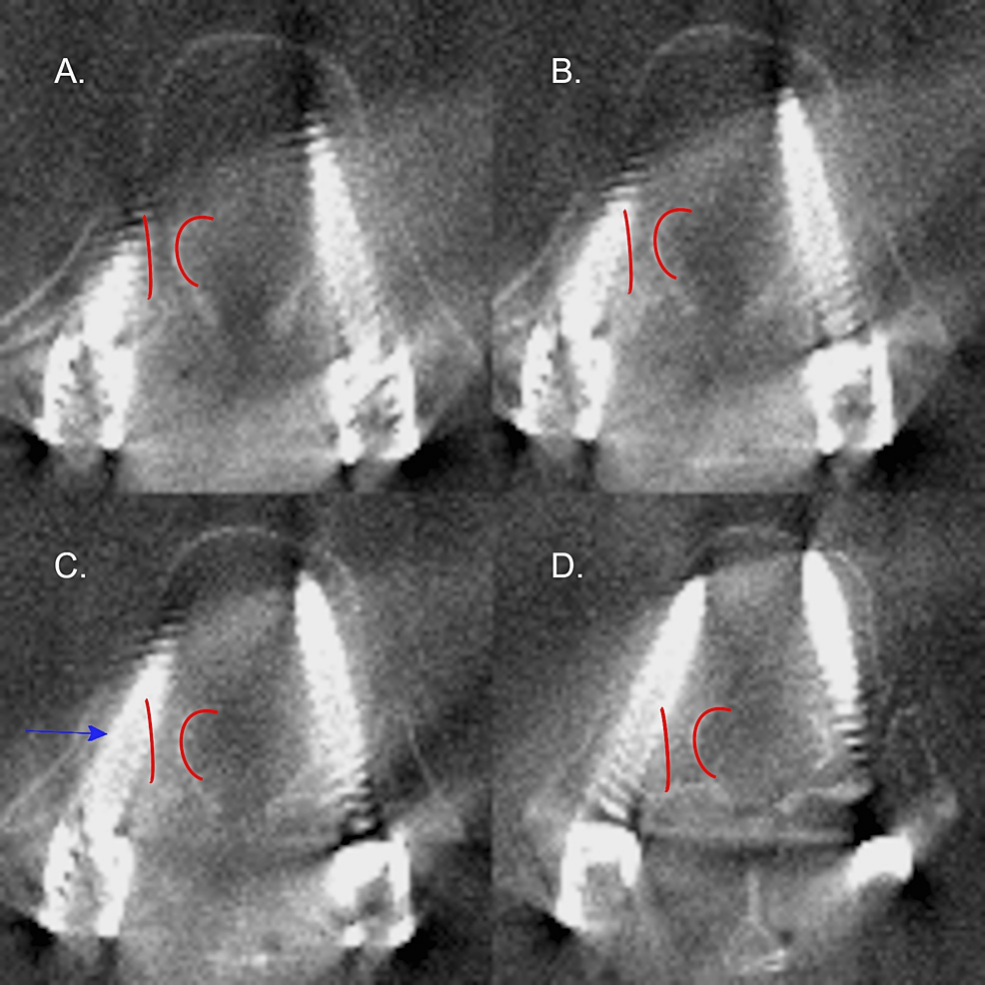
Axial CT radiographs of the right T12 pedicle screw on patient 4. Axial CT radiographs demonstrating the right pedicle screw placement on patient 4 at T12 with an in-out-in (IOI) trajectory. Screw placement progresses from A to D. The pedicle of interest is outlined in red. The blue arrow denotes the largest deviation of the screw. Please note all CT images are in radiographic orientation.

**Figure 3:**
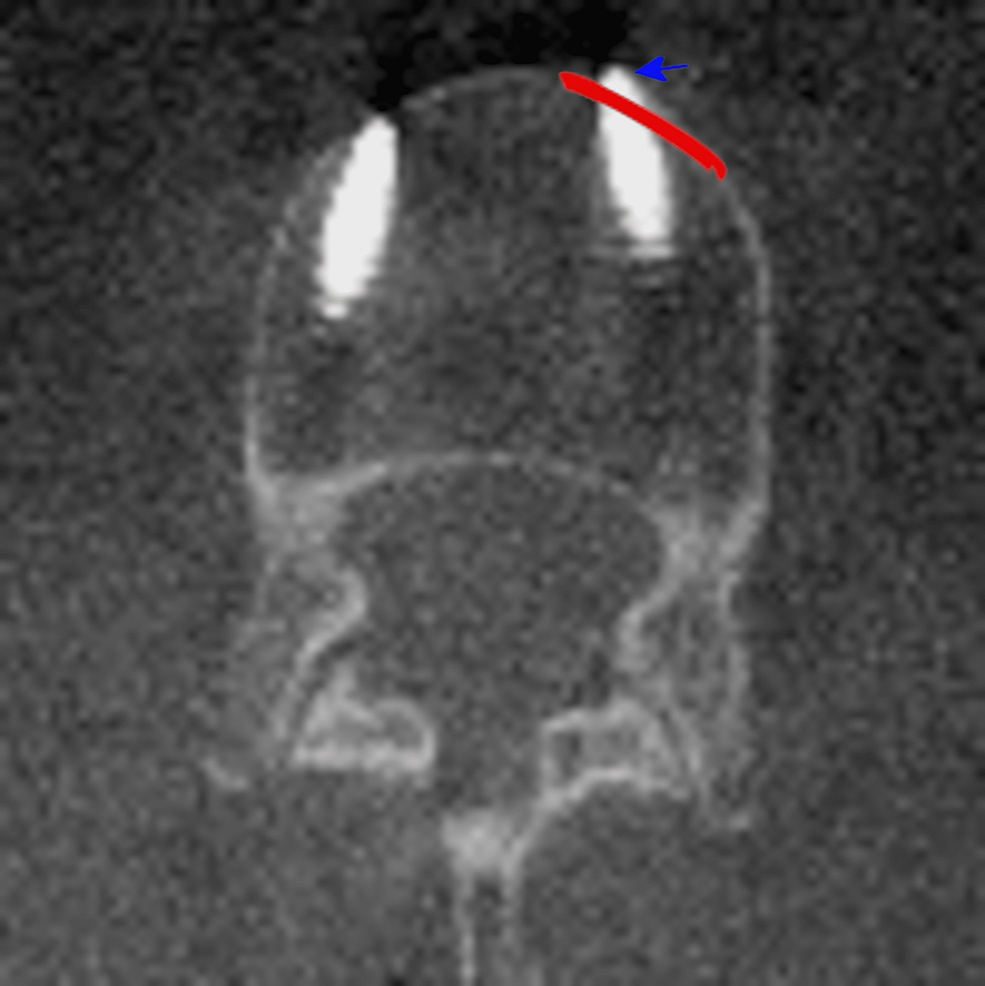
Axial CT radiograph of the left L2 anterior cortex breach on patient 4. An axial CT radiograph of the anterior cortex breach of the left L2 screw on patient 4. The anterior cortex is lined in red. A blue area denotes the screw deviation.

**Figure 4:**
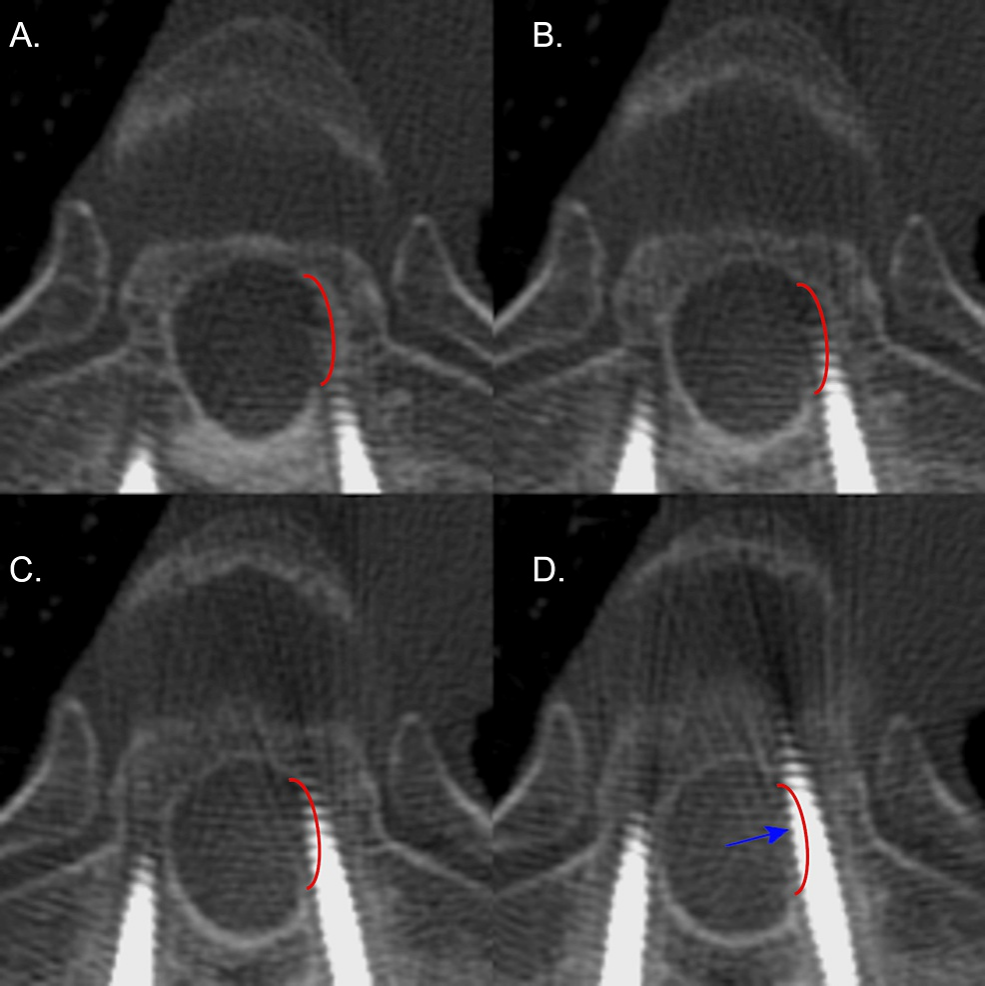
Axial CT radiographs of the left T6 pedicle screw on patient 5. Axial CT radiographs demonstrating the left T6 pedicle screw placement on patient 5 with a medial breach. Screw placement progresses from A to D. The pedicle of interest is outlined in red. The blue arrow denotes the largest deviation of the screw. Please note all CT images are in radiographic orientation.

**Figure 5:**
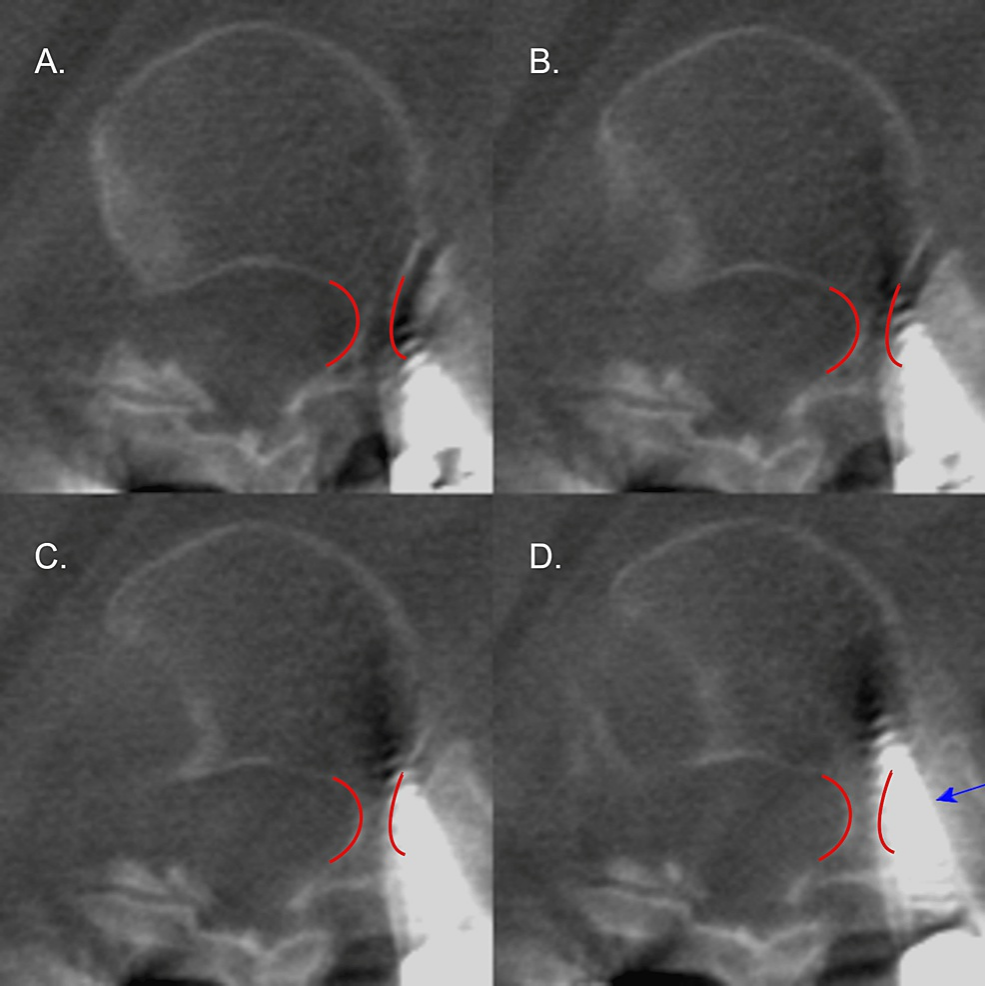
Axial CT radiographs of the left T11 pedicle screw on patient 9. Axial CT radiographs demonstrating the left T11 pedicle screw placement on patient 9 with an in-out-in (IOI) trajectory. Screw placement progresses from A to D. The pedicle of interest is outlined in red. The blue arrow denotes the largest deviation of the screw. Please note all CT images are in radiographic orientation.

**Figure 6:**
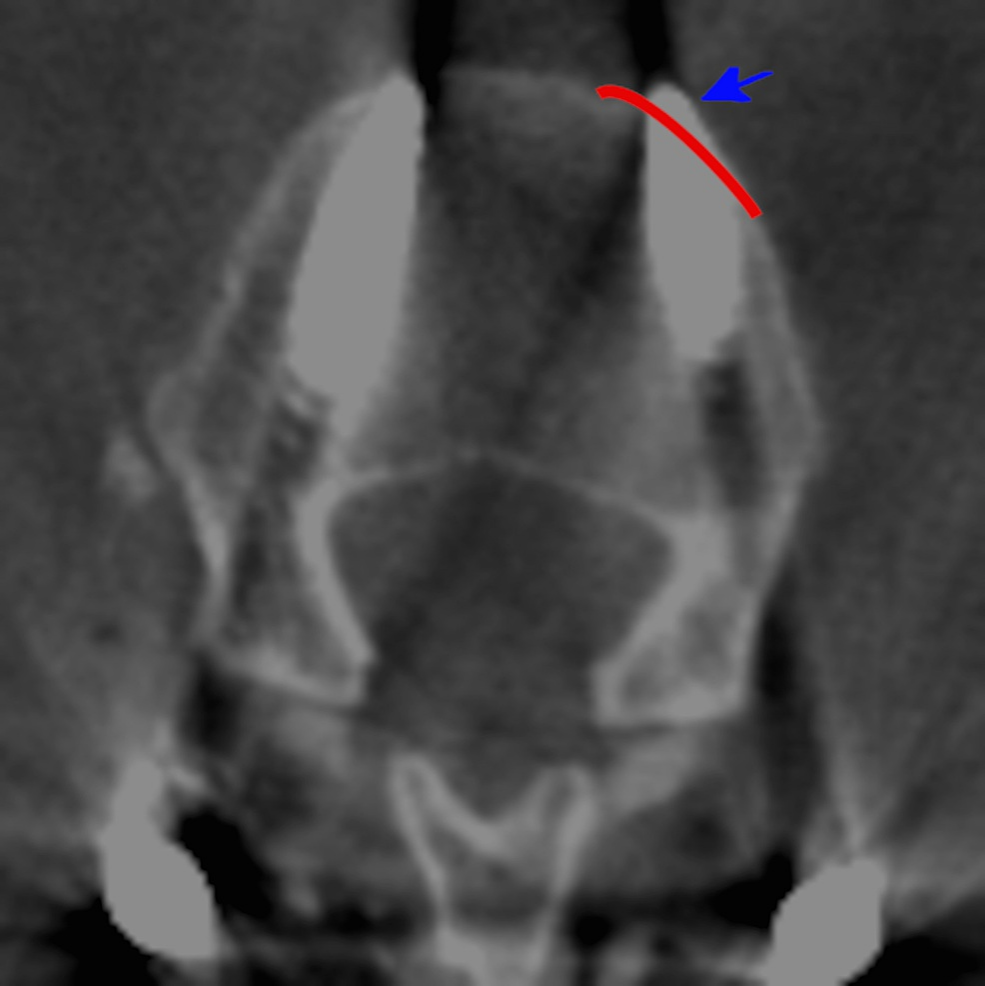
Axial CT radiograph of the left T12 anterior cortex breach on patient 10. An axial CT radiograph of the anterior cortex breach of the left T12 screw on patient 10. The anterior cortex is lined in red. A blue area denotes the screw deviation.

**Figure 7:**
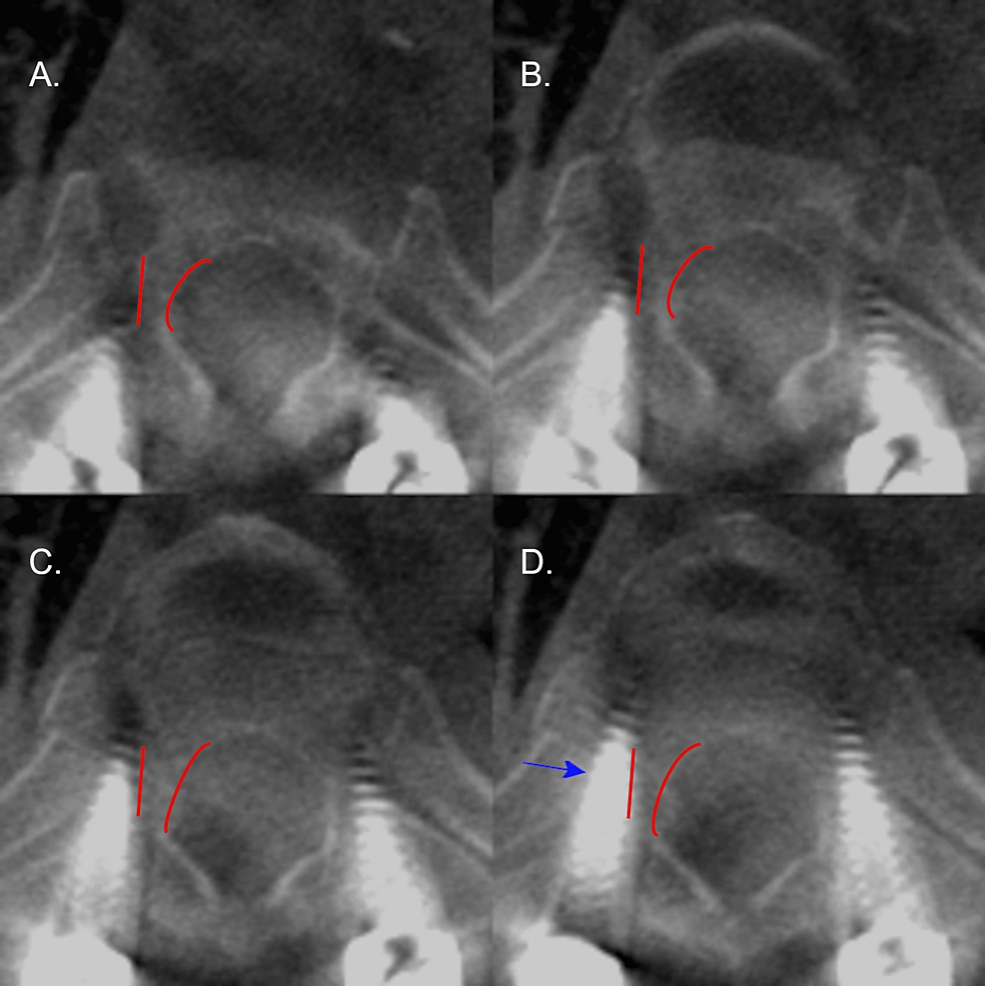
Axial CT radiographs of the right T6 pedicle screw on patient 13 Axial CT radiographs demonstrating the right T6 pedicle screw placement on patient 13 with an in-out-in (IOI) trajectory. Screw placement progresses from A to D. The pedicle of interest is outlined in red. The blue arrow denotes the largest deviation of the screw. Please note all CT images are in radiographic orientation.

**Figure 8:**
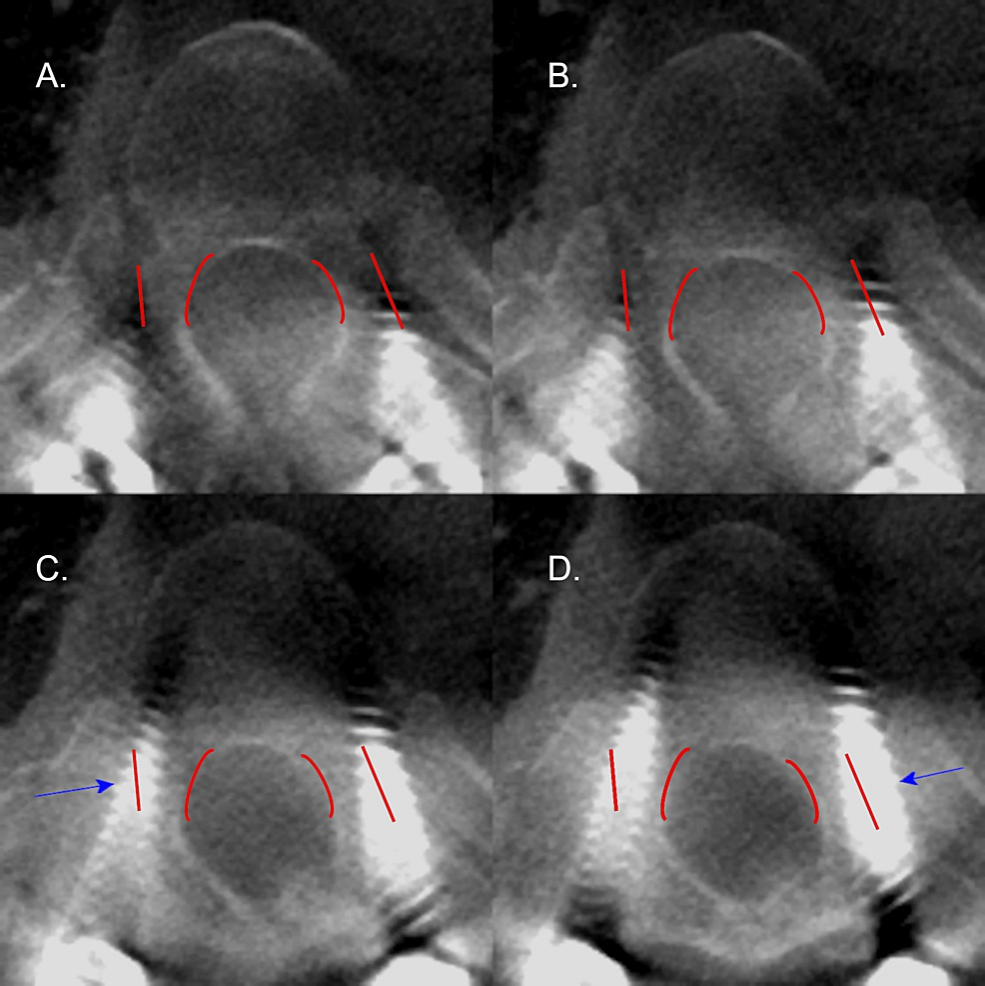
Axial CT radiographs of the T7 pedicle screws on patient 13. Axial CT radiographs demonstrating the T7 pedicle screws on patient 13 with an in-out-in (IOI) trajectory. Screw placement progresses from A to D. The pedicle of interest is outlined in red. The blue arrow denotes the largest deviation of the screw. Please note all CT images are in radiographic orientation.

**Figure 9:**
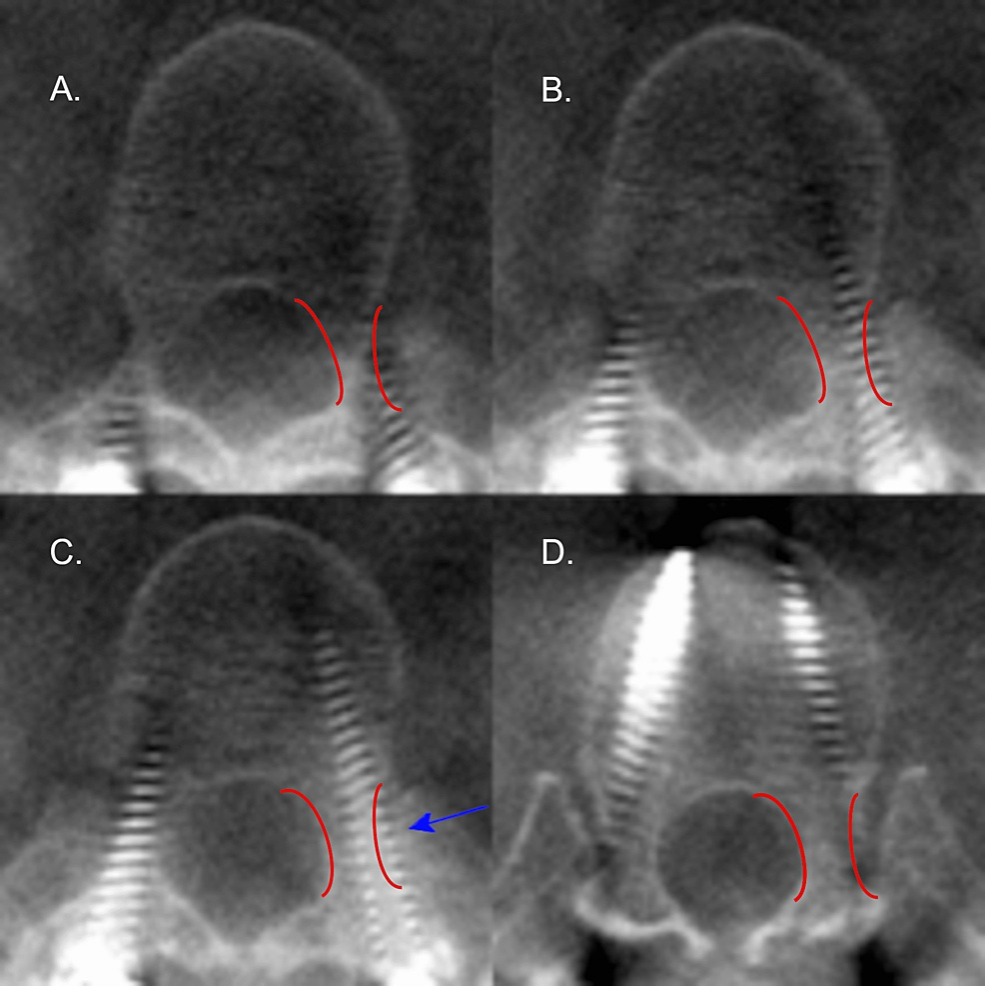
Axial CT radiographs of the left T10 pedicle screw on patient 13. Axial CT radiographs demonstrating the left T10 pedicle screw placement on patient 13 with an in-out-in (IOI) trajectory. Screw placement progresses from A to D. The pedicle of interest is outlined in red. The blue arrow denotes the largest deviation of the screw. Please note all CT images are in radiographic orientation.

Pedicle screw placement was accurate with a <1% (1/126) pedicle breach rate when discounting the pre-planned in-out-in trajectories. Pedicle screw breaches which occurred due to an in-out-in trajectory were noted in Table 1 with “IOI.” Images of each screw breach, as denoted by the neuroradiologists, are provided (Figures 2-9).

**Figure 10:**
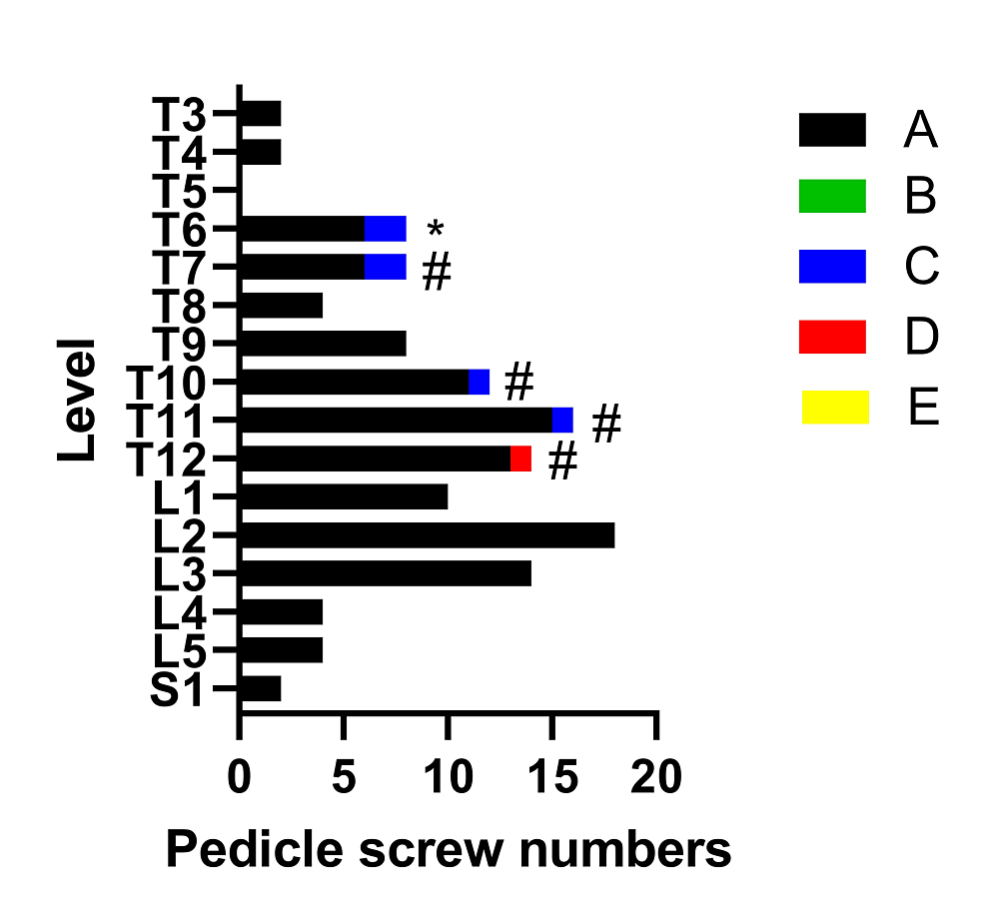
Gertzbein-Robbins screw classification based upon vertebral body level. The number of pedicle screws graphed via the Gertzbein-Robbins classification for each spinal pedicle level. * denotes the true pedicle breach (1 GBS grade C) and a breach due to an IOI trajectory (IOI) trajectory. # denotes all GBS grades > A are due to IOI trajectories.

None of the screws required replacement. The average anterior vertebral body screw breach was 2 mm. The only true pedicle breach (when discounting IOI trajectories) was 2 mm (Figure 4) and was not associated with a change of either neuromonitoring, intraoperatively, or neurological exam, post-operatively. The GRS for each screw was graphed against the vertebral level (Figure 10).

## Discussion

This case series demonstrates the feasibility of utilizing robotic assistance for thoracolumbar pedicle screw placement in acute, traumatic spine surgery. In this paper, after placing 126 pedicle screws in 14 patients we demonstrated the feasibility and safety of robot-assisted spinal fixation. Of the 126 pedicle screws placed, 84 screws were placed from T1-L1 with only a single breach (1.2% breach rate). Our breach rate was comparable to previous studies utilizing O-arm navigation demonstrating the non-inferiority of the robot-assisted pedicle screws in the thoracic spine.

Our secondary findings of this case series include the feasibility of robotic assistance in an acute, traumatic setting. We demonstrated a good safety profile with a single new neurological deficit unrelated to robot use. In fact, three of the four pre-operative neurological deficits improved with surgery. Additionally, blood loss was low (191 mL), and operative times were decreased with a case number.

Pedicle screw placement accuracy is critically important for not only improved fusion rates/strength of the fusion construct but also for patient safety. Previous work has demonstrated that intraoperative navigation improves pedicle screw placement accuracy from 68% to 95% [16]. The addition of a rigid robotic arm further increases pedicle screw placement accuracy to about 99% [14]. However, thoracic pedicle screw placement requires acknowledgment of several anatomical nuances. The sagittal and coronal angulation of the pedicle transitions throughout the thoracic spine. The sagittal pedicle angle increases from 0 degrees at T1 to 10 degrees at T8. The sagittal pedicle angle decreases from T8 to 0 degrees at T12 [8]. In the coronal plane, pedicle angulation decreases from 10-15 degrees at T1 to 5 degrees at T12 [8]. Additionally, the transverse process, an anatomic landmark for the lumbar pedicle, does not align with the thoracic pedicle in the axial plane. The transverse process progresses from the rostral to the pedicle at T1 to the caudal to the pedicle at T12 with the T6 or 7 transverse processes aligning with the pedicle [8]. The morphing anatomical landmarks throughout the thoracic spine creates a scenario ideal for navigation as navigation provides an additional check to known anatomical landmarks. In this study, robot-assisted pedicle screw placement was accurate in the thoracic spine with a breach rate of 1.2% without any new neurological deficits. While <2% of screws were not in the optimal trajectory, the screws did not need to be replaced, and no issues were caused by the alternative trajectories. This finding is comparable to previous work which has shown that neurovascular compromise occurs in 4.2% of patients [17]. Additionally, not requiring replacement of the screw is important as Definoet al. found insertion, removal and reinsertion of a pedicle screw in the same hole compromised [18] resistance to pullout. Our results support the use of robot-assisted navigation in the thoracic region and the non-inferiority of robot-assisted navigation to other screw-placement methods due to the superb accuracy and lack of neurological deficits.

Additionally, our study is unique as the cases occurred in an acute, traumatic setting. Acute, spine surgery provides unique challenges which are nonmodifiable including increased likelihood of hypotension from shock, increased neurological complications, increased operative hemorrhages, polytraumas, and operating in non-ideal conditions despite operative methods [19]. Understanding the feasibility of robot-assisted spine surgery is important as research on spinal cord injuries suggests hyperacute (<8 hours post-injury) decompression should be performed. Jug et al. and Grassner et al. demonstrated improved neurological outcomes for hyperacute (<8 hours post-injury) versus acute surgery (9-24 hours post-injury) [12,13]. Therefore, choosing a method with superb accuracy, efficiency, and reliability is fundamental to successful surgery.

The robotic arm is especially useful in an acute, traumatic setting due to the rigidity and additional support provided to instruments. In unstable spines, touching the instruments to a fractured vertebra may displace the bony elements and lead to undesirable trajectories. However, with additional aids such as the robotic arm, such outcomes are less likely. Furthermore, swelling can lead to distortion of landmarks, which inhibits independent verification of landmarks, which can be partially mitigated by dual point verification in robotic spine surgery. However, despite robot-assisted spine surgery having many advantages, no work has demonstrated the feasibility of robotic-assisted surgery with a focus on either the thoracic spine or acute, traumatic surgery. A review of previous studies for robot-assisted pedicle screw placement is heavily biased in two ways: (1) to L4-S1 and (2) to elective cases. A study by Keric et al., one of the largest robotic pedicle screw placement studies with 2,067 pedicle screws, had 321/406 (79%) cases focused on the lumbar/lumbosacral or lumbosacral area [9]. However, the overrepresentation of lumbar cases is not isolated to this study. A study by Fan et al. published a paper on robot-assisted pedicle screw placement with posterolateral interbody fusion. In this study, 54 of the 83 patients had screws placed at T11 or below [10]. Meanwhile, another study by Devito et al. did not publish the levels that were instrumented limiting the conclusions which can be drawn [11]. This study demonstrates that robot-assisted pedicle screws in the thoracolumbar spine during acute, traumatic surgery are both feasible and accurate which is in agreement with previous studies demonstrating the accuracy of robot-assisted pedicle screws [20-22]. However, a large, randomized trial should be performed to confirm or disprove the superiority of robot-assisted pedicle screw placement as compared to pedicle screws placed with other techniques in the thoracic spine during an acute, traumatic setting.

The accuracy of our pedicle screw placement can be traced to our meticulous attention to merge scores and stabilization of instruments via the robotic arm so as not to deform the spine while placing screws. Specifically, we found the instability of the spine can lead to motion of the spinal column even during breathing if the fracture is severe. Spinal instability would lower the accuracy of our pre-operative and interoperative image merge. To help address a proper merge, we have found breath holds were crucial. For improved screw accuracy, we found apnea before initial drilling and screw placement, stabilization of instrumentation by a robotic arm, and minimizing pressure on unstable areas when drilling as methods to maximize accuracy. Using these steps, we have managed to have accurate screw placements in an acute, traumatic setting. Also, it is important to note the dual verification required the placement of a second array in the pelvis during surgery. When performing navigated screws, the distance from the array can lead to increased variability in infrared detection. However, the dual array utilized in this study had no issues.

One major critique of this paper may be the increased operative time associated with utilizing a robot. However, we do not discuss additional procedures being performed such as laminectomies which were included in our operative time. Furthermore, we demonstrated that operative times are continuing to improve as no plateau has been met (Figure 1). These findings are consistent with previous adoptions of 3D-navigated techniques in spine surgery [23,24]. Optimization will likely decrease operative times further. Interestingly, previous studies have suggested that robot-assisted surgery has a small learning curve [14]. Through robot-assisted surgery, more confidence may be placed on pre-planned trajectories allowing the surgeon to focus on the additional procedures such as corpectomies or laminectomies as well as provide further confidence in the navigation necessary for the thoracic spine.

The second critique of this paper may be the planned IOI trajectories. Intuitively, if one is placing pedicle screws with robotic assistance, each screw should be wholly within the pedicle. However, nuances in anatomy make this difficult. When placing pedicle screws in the thoracic spine, some pedicles are smaller than the standard smallest screw diameter. Furthermore, when placing screws optimally, not all screws can be wholly in the pedicle and allow for attachment to the rod despite the polyaxial heads. The surgical goal in regard to pedicle screw trajectory was to optimize the trajectory while enabling feasible direct attachment to the rods. Therefore, after taking into account both pedicle size and rod attachment, IOI trajectories were at times a necessity.

Another critique of this paper would be the blood loss. Previous work has demonstrated decreased blood loss for robot-assisted spinal surgery as opposed to freehand techniques [22]. However, our surgeries were performed in the acute phase and in the context of other injuries. Therefore, direct comparisons for blood loss, specifically, are less meaningful and misleading when compared to historical studies. While we agree that decreased blood loss is interesting, being a case series and lacking a control cohort, this study does not provide the correct framework to evaluate blood loss, and a future study would be needed to address this question. Furthermore, this study may be critiqued for the hospitalization length of the patients. However, given that each patient is a polytrauma, the hospitalization duration comparison to previous studies loses meaning as complicating injuries contributed to the duration of the hospitalization. For instance, one patient was hospitalized over two months for non-spine-related injuries and complications.

This study does have caveats. This study had a low number of cases. Therefore, as with any technology, we cannot state that robotic-assisted spine surgery would be appropriate in all acute, spinal trauma cases and can only claim noninferiority versus other methods. However, given that our goal was proof of concept, we believe that despite our low number of cases, it provides valuable insight for thoracic spine surgery during the acute, traumatic setting.

A second caveat may be that spine robots are expensive and cost-prohibitive for a number of hospitals [25]. However, the purpose of this paper was not to address affordability, and this issue has been previously documented. Finally, this study was performed by a single surgeon at a single institution, and the argument could be made that these results are not applicable to multiple surgeons. However, there was some variability in surgeons as the accompanying resident-assistant varied between cases.

## Conclusions

Robot-assisted pedicle screw placement is a feasible alternative to other pedicle screw placement techniques in thoracic and lumbar spine fixation surgery during an acute, traumatic setting with many benefits. These benefits include multiple redundancy checks for screw accuracy, improved stability of instruments, and less deformity of fractured vertebrae due to the rigid robot arm supporting the tools. Additionally, we demonstrated the operative times decrease as the surgeon gains experience. Furthermore, we demonstrate safe pedicle screw placement in both the lumbar and thoracic spine. We hope through the adoption of robot-assisted spine surgery in the thoracic and lumbar region during the acute, traumatic setting, patients will have the safest surgeries possible.
